# Preoperative Ultrasonographic Evaluation for Malignancy of Soft-Tissue Sarcoma: A Retrospective Study

**DOI:** 10.2174/1874325001812010075

**Published:** 2018-03-16

**Authors:** Takeshi Morii, Tomonori Kishino, Naoko Shimamori, Mitsue Motohashi, Hiroaki Ohnishi, Keita Honya, Takayuki Aoyagi, Takashi Tajima, Shoichi Ichimura

**Affiliations:** 1 Department of Orthopaedic Surgery, Faculty of Medicine, Kyorin University, 6-20-2 Shinkawa, Mitaka, Tokyo 181-8611, Japan; 2 Department of Laboratory Medicine, Faculty of Medicine, Kyorin University, 6-20-2 Shinkawa, Mitaka, Tokyo 181-8611, Japan; 3 Department of Medical Radiological Technology, Faculty of Health Sciences, Kyorin University, 5-4-1 Shimorenjaku, Mitaka, Tokyo 181-8612, Japan

**Keywords:** Soft tissue tumor, Diagnosis, Ultrasonography, High-grade sarcoma, Low-grade sarcoma, Malignancy

## Abstract

**Background:**

Ultrasonography is useful for distinguishing between benign and malignant soft-tissue tumors. However, no study has focused on its usefulness in the differential diagnosis between low-grade and high-grade soft-tissue sarcomas. We conducted a retrospective study to determine the usefulness of the parameters of ultrasonograph and to develop a practical scoring system for distinguishing between high-grade and low-grade sarcomas.

**Methods:**

Twenty-two cases of low-grade and 43 cases of high-grade malignant soft-tissue sarcoma were enrolled. Ultrasonography parameters including the longest diameter, depth of the tumor, echogenicity, tumor margin, and vascularity defined according to Giovagnorio’s criteria were analyzed as factors to distinguish between the two types of sarcoma. Significant factors were entered into a multivariate model to define the scores for distinction according to the odds ratio. The usefulness of the score was analyzed *via *receiver operating characteristic analyses.

**Results:**

In univariate analysis, tumor margin, echogenicity, and vascularity were significantly different between low- and high-grade sarcomas. In the multivariate regression model, the odds ratio for high-grade vs. low-grade sarcoma was 8.8 for tumor margin, 69 for echogenicity, and 8.3 for vascularity. Scores for the risk factors were defined as follows: 1, ill-defined margin; 2, hypoechoic echogenicity; and 1, type IV in Giovagnorio’s criteria. The sum of each score was confirmed by receiver operating characteristic analysis. The area under the curve was 0.95, with a cut-off score of 3, indicating that the scoring system was useful.

**Conclusion:**

The ultrasonography parameters of tumor margin, echogenicity, and vascularity are useful for distinguishing between low- and high-grade sarcomas.

## 1 INTRODUCTION

Soft-tissue sarcoma is a rare neoplasm of mesenchymal origin. As biological properties, differentiation, histological origin, local invasiveness, sensitivity to radiotherapy and chemotherapy, and incidence of local recurrence and metastasis vary significantly, its treatment should be selected on the basis of the nature of the tumor and should be decided on a case-to-case basis [1-4]. The treatment modality is selected based on the histological grade of the tumor determined *via *pathological examination of the specimen from a lesion, which is a representative parameter of tumor activity. The Fédération Nationale des Centres de Lutte Contre le Cancer (FNCLCC) grading system is used for soft-tissue sarcomas defined by tumor differentiation, mitosis count, and tumor necrosis; this system is commonly the basis for making decisions about the use of chemotherapy and definition of surgical margins [5]. For example, neoadjuvant and adjuvant chemotherapy are used only for high-grade sarcoma (FNCLCC grade II and III) under the hypothesis that therapeutic reagents are effective only for sarcoma cells with a high mitotic rate [1]. On the other hand, conservative surgery with a closer surgical margin is used for low-grade sarcomas, as they show less local invasiveness [2, 3]. Thus, the grading of sarcomas is critical in the management of soft-tissue tumors. Importantly, grading without the need for invasive biopsy could help in the management of soft-tissue sarcoma.

As the malignancy of the sarcoma is closely related to the biological behavior of tumor cells, including high growth ability represented by a high mitotic rate, upregulated metabolism, and inhomogeneous component caused by central necrosis, the histological grade could be determined *via *indirect and less-invasive methods that can detect the abnormal biological behavior of tumor cells. For example, Folpe *et al* used fludeoxyglucose positron emission tomography (FDG-PET) to predict the grading of sarcomas; this modality can detect the upregulated metabolism of tumor cells [6]. Similarly, Gruber *et al* reported that the inhomogeneous enhancement pattern of magnetic resonance imaging (MRI), a standard radiological modality for evaluating soft-tissue tumors, was useful for determining the grades of soft-tissue tumors [7].

Ultrasonography (US) is an imaging modality widely used for the evaluation of soft-tissue tumors. It has several advantages over FDG-PET and MRI: it is concise, is economical for private clinics, does not require injections, is less invasive, and is easy to perform on children [8, 9]. US is useful for detecting soft-tissue tumors, specifically for examining its location; shape; margin; size; water component; and condition of vessels including the volume of blood flow, vessel density, and structural abnormality without injection [10-16]. In addition, several studies have reported that US is useful for the specific diagnosis of soft-tissue tumors such as neurofibromas [17], dermatofibrosarcoma protuberance [18], synovial sarcoma [19] and well-differentiated liposarcoma/atypical lipomatous tumors [20]. Several previous studies reported that tumor characteristics such as large size, infiltration into the surrounding tissues, inhomogeneous enhancement, abnormal vasculature morphology, upregulated vascularity, and upregulated blood flow are useful for distinguishing between benign and malignant soft-tissue tumors (Table **1**), [7, 10-14, 16, 21-26]. In our previous series, we reported that the maximum size, tumor margin, and vascularity evaluated using US were extracted as significant properties of the malignant soft-tissue tumor [7]. However, to our knowledge, US has not yet been used for grading soft-tissue sarcomas so far. Assuming that tumor malignancy is represented by the above-mentioned biological behaviors, the significant parameters for distinguishing between benign and malignant soft-tissue tumors may be applicable for distinguishing between high-grade and low-grade soft-tissue sarcomas. Therefore, in the present study, we aimed to determine the usefulness of US parameters except for the Sonazoid-enhancement pattern, which is not available for soft-tissue tumors in Japan and to develop a practical scoring system for distinguishing between high-grade and low-grade soft-tissue sarcomas.

## 2 MATERIALS AND METHODS

To evaluate the value of US parameters to the differential diagnoses of low-grade and high-grade sarcomas, this retrospective uncontrolled study examined data from the medical records of patients with sarcomas. The inclusion criteria were as follows: pathological diagnosis of primary soft-tissue sarcoma; surgical treatment at the authors’ institutions between January 2013 and December 2015; performance of preoperative US examination before any intervention including biopsy; and availability of clinical, pathological, and radiological data. A total of 65 patients (30 men, 35 women) with soft-tissue sarcomas were enrolled. The mean and median ages were 61 years and 64 years, respectively. The pathological diagnoses were as follows: liposarcoma in 30 cases, undifferentiated pleomorphic sarcoma in 14 cases, myxofibrosarcoma in 5 cases, malignant peripheral nerve sheath tumor in 5 cases, leiomyosarcoma in 2 cases, dermatofibrosarcoma protuberance in 2 cases, and others in 7 cases. Cases were classified according to the FNCLCC grading system [5] as follows: grade I, 22 cases; grade II, 15 cases; and grade III, 28 cases. In the present study, grade I was defined as a low grade, and grades II and III were defined as high grades (Table **2**).

The US was performed before any intervention, including open biopsy, radiotherapy, chemotherapy, and resection, by using an Aplio 500 ultrasound scanner (Toshiba Medical Systems, Tochigi, Japan). All US examinations were performed using linear (10 MHz) and convex (3.5 MHz) transducers.

**Fig. (1):**
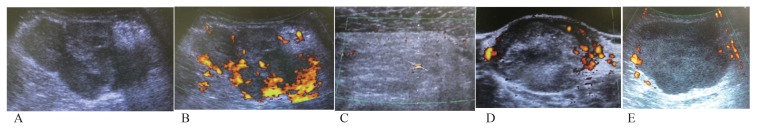
Representative ultrasonography findings of patients in this study. Grade III undifferentiated pleomorphic sarcoma in the thigh (67-year-old man). Hypoechoic, ill-defined, infiltrating margin (**A**) with prominent vascularization (Giovagnorio’s criteria type VI) (**B**). A score of 4 is assigned, indicating high-grade tumor (true positive). (**C.**) Grade I well-differentiated liposarcoma in the buttock (52-year-old man). Hyperechoic mass and undefined margin with limited marginal vascularity (Giovagnorio’s criteria type III). A score of 1 is assigned, indicating low-grade tumor (true negative). (**D.**) Grade I dermatofibrosarcoma protuberance in the shoulder (34-year-old man). Hyperechoic, well-defined margin with prominent vascularization (Giovagnorio’s criteria type VI). A score of 1 is assigned, indicating low-grade sarcoma (true negative). (**E.**) Grade III malignant peripheral nerve sheath tumor in the abdomen wall (74-year-old man). Hypoechoic, well-defined margin with limited marginal vascularity (Giovagnorio’s criteria type III). A score of 2 is assigned, indicating low-grade sarcoma (false negative).

We selected several independent variables evaluated *via* US, such as the longest diameter, depth of the tumor, echogenicity, tumor margin, and tumor vascularity, which are significant factors in distinguishing between benign and malignant soft-tissue tumors [10, 12-14, 16, 21]. The gray-scale US was used to evaluate the longest diameter, depth of the tumor (deep/subcutaneous), echogenicity, and tumor margin. Echogenicity was defined as hypoechoic or hyperechoic/isoechoic, relative to the adjacent muscle tissue [10, 21]. Tumor margins were defined as per previous reports: well-defined (clear-cut and thin, capsule-like) or ill-defined (uncertain margin with respect to adjacent normal tissue or certain irregular margin with respect to adjacent normal tissue and wider transitional zones) (Fig. **1**) [10, 11]. Doppler sonography was used to evaluate tumor vascularity based on Giovagnorio’s criteria [13]. In brief, the vascularity patterns were classified as avascular (type I), hypovascular with a single vascular pole in the hilum (type II), hypervascular with multiple peripheral poles (type III) (Figs. **1C** and **1E**), or hypervascular with internal vessels (type IV) (Figs. **1A**, **1B** and **1D**). In the present study, the vascularity pattern was considered a continuous variable. Gray-scale US in combination with Doppler US was prospectively performed by either one of two investigators (N.S. and M.M.), who were blinded to the patient data and final histological diagnosis.

The chi-squared, Fisher's exact, and Mann–Whitney *U*-tests were used in the univariate analyses for comparing each parameter between the low-grade and high-grade groups. The cut-off values were evaluated using receiver operating characteristic (ROC) analyses, with the significant factors included as continuous variables. Variables that were significant in univariate analyses (p < 0.05) were entered into a multivariate logistic regression model. Based on the odds ratio determined by the regression model, we established a scoring system to distinguish between the low-grade and high-grade groups. Statistical analyses were performed using JMP software (version 10; SAS Institute Inc., Cary, North Carolina, USA). All procedures were performed in accordance with the ethical standards of the responsible committee on human experimentation (institutional and national) and the Helsinki Declaration of 1964 and later versions. The study was approved by the institutional review board of the authors’ institution.

## 3 RESULT

**Fig. (2):**
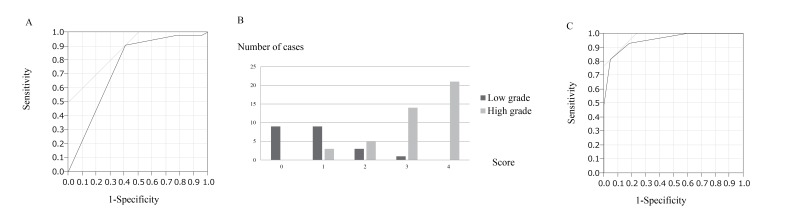
**A.** Receiver operating characteristic (ROC) curve for the cut-off value for Giovagnorio’s criteria to distinguish between low-grade and high-grade sarcomas. **B.** Distribution of low-grade and high-grade cases according to the score. **C.** ROC curve to determine the cut-off score for low-grade and high-grade sarcomas.

The longest diameter, depth of the tumor, tumor margin, echogenicity, and vascularity evaluated using US were compared between the low-grade and high-grade groups *via *univariate analysis. The results showed that tumor margin (p = 0.005), echogenicity (p < 0.0001), and vascularity (p < 0.0001) were significantly different (Table **3**) between the two groups. ROC analysis performed to determine the most useful cut-off value of Giovagnorio’s criteria for distinguishing between the two groups showed that a cut-off value of 4 was the most appropriate (Fig. **2A**), with a sensitivity and specificity of 0.91 and 0.41, respectively. The area under the curve of 0.75 indicated that the cut-off value was useful for determining the malignancy of the tumor.

The three significant parameters were subsequently entered into a logistic regression model. Multivariate analysis showed that the three variables (margin, echogenicity, and vascularity) were independent risk factors (Table **4**). The odds ratio for high-grade vs. low-grade sarcomas was 8.8 for tumor margin, 69 for echogenicity, and 8.3 for vascularity. Based on these ratios, we established a scoring system to distinguish between low-grade and high-grade sarcomas according to the US findings (Table **5**). The prognostic score was calculated by adding all the scores of individual factors. Each case was scored from 0 to 4 points (Fig. **2B**). The scores of low-grade and high-grade sarcomas (mean ± standard deviation) were 0.8 ± 0.8 and 3.2 ± 0.9, respectively. Most cases of low-grade sarcomas were scored <2, whereas most cases of high-grade sarcomas were scored >3. The scores of high-grade sarcomas were significantly higher than those of low-grade sarcomas (p < 0.0001). Finally, we plotted the ROC curve for this model (Fig. **2C**). The cut-off value of the score to distinguish between low-grade and high-grade sarcomas was determined to be 3, with a sensitivity and specificity of 0.81 and 0.95, respectively. The area under the curve of 0.95 indicated that the cut-off was useful for determining malignancy of the tumor in terms of tumor margin, echogenicity, and vascularity.

## 4 DISCUSSION

In the present study, we demonstrated the usefulness of US for differential diagnosis between low-grade and high-grade soft-tissue sarcomas. In addition, three significant factors were found to be useful for this distinction: tumor margin, echogenicity, and vascularity.

Chou *et al* defined ill-defined and infiltrated margins as uncertain margins with respect to adjacent normal tissue and certain irregular margin with respect to adjacent normal tissue and wider transitional zone, respectively. These parameters were useful for distinguishing between certain malignant soft-tissue tumors from benign lesion [11]. Similarly, Oebisu *et al* defined ill-defined margins as uncertain margins with respect to adjacent normal tissue and reported that the frequency of ill-defined margin was significantly higher in malignant soft-tissue tumors than in benign lesions [10]. The infiltration trend was histologically confirmed to be the characteristic of malignant soft-tissue tumors. In MRI, the infiltration pattern of expanding along with the fascia or neurovascular or musculature plane around the soft-tissue sarcoma is known as “tail-like pattern”, and this abnormal shadow was pathologically proven to be infiltrating viable cells or edematous change [27, 28]. Even if the “tail-like pattern” is not seen around sarcomas, viable tumor cells are frequently seen outside the margin of the tumor mass [28]. Considering that benign soft-tissue tumors rarely involve local recurrence with intralesional or marginal resection, infiltration is a universal characteristic of malignant soft-tissue sarcoma. Although histological confirmation of the infiltration pattern on US is needed, an ill-defined margin in US can be considered to represent the invasiveness of malignant soft-tissue sarcoma.

Upregulated vascularity is also a universal characteristic of malignant tumors [13, 29]. Upregulated metabolism in the process of non-physiological cell proliferation causes hypoxic conditions around the tumor, resulting in the activation of hypoxia-inducible factor 1 (HIF-1), a transcription factor that is critical in the adaptive cellular response to hypoxia. HIF-1 activates several intracellular signaling pathways for cellular metabolism, angiogenesis, proliferation, and survival by activating related genes including vascular endothelial growth factor (*VEGF*), which promote tumor angiogenesis [30]. As *VEGF* promotes many aspects including an increase in the number of vessels and structural abnormalities, variation in caliber, a non-hierarchical network, lack of smooth muscle cells, disturbed pericapillary architecture, and incomplete vessel walls can be seen in non-physiological tumor angiogenesis [31]. US can easily and non-invasively detect such abnormalities in angiogenesis in the tumor, and therefore, it is broadly applied for distinguishing malignant soft-tissue tumors from benign lesions [10, 12-14, 16, 21, 22]. Moreover, in the previous report, we hypothesized that intraoperative blood loss during resection of malignant soft tissue tumor could be predicted using blood flow parameters evaluated *via *US, and showed that vessel density and time-averaged flow velocity could predict the need for intraoperative blood transfusion [32]. However, in terms of grading sarcomas, immunohistochemical studies for markers of vessels or proteins of pro-angiogenetic markers, such as HIF-1 and VEGF, or markers for a hypoxic condition such as carbonic anhydrase 9 or glucose transporter-1 [33] have been reported to be useful, rather than US. The present study aimed to determine whether US can be used for distinguishing between low-grade and high-grade sarcomas by examining the differences in their biological properties. Our results showed that, at least with respect to the angiogenesis status, immunohistochemical analyses compliment the capability of US, thereby supporting our results.

The usefulness of echogenicity, which is one of the significant factors for distinguishing between low-grade and high-grade sarcoma in the present study, for distinguishing between benign and malignant soft-tissue tumors is controversial. Nagano *et al* reported that low echogenicity was a significant characteristic of malignant soft-tissue tumors [12], whereas Oebisu and Chous reported that echogenicity was not a useful parameter for this distinction [10, 11]. Futani *et al* aimed to differentiate between lipoma and well-differentiated liposarcoma (WDLS), and reported that although the distinction was possible by evaluating the angiogenic conditions using Doppler US, no difference in gray-scale findings including echogenicity between the lipoma and WDLS was confirmed [20], suggesting that non-hypoechoic echogenicity was a common finding in both lipoma and low-grade WDLS. As such, hypoechoic echogenicity is a specific property of high-grade sarcoma rather than malignant soft-tissue sarcoma. In the present study, 16 of the 17 WDLS cases, and all 2 dermatofibrosarcoma protuberance cases, which is another representative low-grade sarcoma, did not show hypoechoic echogenicity.

The most critical limitation of the present study is that the result of the grading system is disease dependent, *i.e.* the distinction between low-grade and high-grade sarcomas with the same histological diagnosis was not confirmed. A large proportion of low-grade sarcomas are WDLS. Thus, our results may have been from the specific properties of WDLS in US findings, rather than those with low-grade sarcomas. Therefore, a large number of cases of specific histological subtypes with different grades are needed in the future. In addition, future studies should aim to determine what ill-defined margins or hypoechoic echogenicity represent in biological processes. Considering the infiltrating trend as a universal characteristic of malignancy and upregulated frequency of ill-defined margin in malignant tumors, particularly in high-grade sarcomas, an ill-defined margin might reflect an invasive process, but this assumption lacks histological confirmation. Similarly, the biological process underlying hypoechoic echogenicity should be determined in the future.

## CONCLUSION

Distinction between high-grade and low-grade sarcoma is possible using US, considering the following parameters: tumor margin, echogenicity, and vascularity.

## Figures and Tables

**Table 1:** Previous reports of differential diagnosis between benign and malignant soft tissue tumors.

Author (Year of publication)	Significant parameters useful for discrimination	Reference
Giovagnorio F (1999)	Increased vascularization	[13]
Belli P (2000)	Irregular margin, Hypoechoic pattern, Increased vascularization, Vessel arrangement, Systolic velocities	[21]
Bodner G (2002)	Vessel arrangement, Vessel structure, Minimum/maximum resistive index	[14]
Griffith JF (2004)	Vascular organization, End diastolic velocity, Resistive index	[16]
Chiou HJ (2009)	Infiltrated margins, Scalloped shape, Size, Ill-defined margin,	[11]
Chen CY (2009)	Morphologic and texture feature diagnosed by computer-aided diagnosis system	[22]
Chiou HJ (2010)	Vascular index, Flow index, Vascular-flow index	[23]
Stramare R (2013)	Peak enhancement intensity	[24]
Oebisu N (2014)	Size, Depth, Heterogeneous texture, Ill-defined margin, Increased vascularization	[10]
Nagano S (2015)	Size, Hypoechoic pattern, Heterogeneous texture, Increased vascularization,	[12]
De Marchi A (2016)	Inhomogeneous perfusion, Arterial uptake	[25]
Gruber L (2016)	Inhomogeneous contrast enhancement	[7]
Morii T (2018)	Size, Ill-defined margin, Increased vascularization	[26]

**Table 2:** Histological diagnosis of the subjects.

Diagnosis	Low grade	High grade	Total
LS	18	12	30
WDLS	17	0	17
Myxoid LS	1	6	7
Pleomorphic LS	0	1	1
Dedifferentiated LS	0	5	5
UPS	0	14	14
Myxofibrosarcoma	0	5	5
MPNST	0	5	5
Leiomyosarcoma	0	2	2
DFSP	2	0	2
Others	2	5	7
Total	22	43	65

LS, liposarcoma; WDLS, well-differentiated liposarcoma; UPS, undifferentiated pleomorphic sarcoma; MPNST, malignant peripheral nerve sheath tumor; DFSP, dermatofibrosarcoma protuberance.

**Table 3:** Results of the univariate analyses of ultrasonography parameters for comparing low-grade and high-grade sarcomas.

Findings	Low grade	High grade	p value
The longest diameter (mm)	118 ± 71*	107 ± 46*	0.80
Depth			0.57
Subcutaneous	6	15	
Deep	14	21	
Margin			0.005
Well-defined	17	17	
Ill-defined	5	26	
Echogenicity			<0.0001
Hypoechoic	2	37	
Iso/Hyperechoic	20	6	
Vascularity			
As continuous variable	3.1 ± 0.9*	3.9 ± 0.5*	<0.0001
Type I	1	1	<0.0001
Type II	4	0	–
Type III	8	3	–
Type IV	9	39	–

*, mean ± standard deviation

**Table 4:** Results of multivariate analyses of ultrasonography parameters for comparing low-grade and high-grade sarcomas.

Findings	p value	Odds ratio	95% CI
Margin	–	–	–
Well-defined	–	Reference	–
Ill-defined	0.02	8.8	1.4–87
Echogenicity	–	–	–
Iso/Hyperechoic	–	Reference	–
Hypoechoic	< 0.0001	69	11–856
Vascularity	–	–	–
Type I/II/III	–	Reference	–
Type IV	0.02	8.3	1.3–74

CI, confidence interval

**Table 5:** Scoring system based on the odds ratios in multivariate analysis.

Independent risk factor	Score
Margin	–
Well-defined	0
Ill-defined	1
Echogenicity	–
Iso/Hyperechoic	0
Hypoechoic	2
Vascularity	–
Type I/II/III	0
Type IV	1
